# A Comparison of Two Transport Monitor Systems With Regard to Efficiency and Staff Satisfaction in the Perioperative Setting

**DOI:** 10.7759/cureus.60481

**Published:** 2024-05-17

**Authors:** Kevin M Tourelle, Jonas Fricke, Manuel Feißt, Maik von der Forst, Maximilian Dietrich, Daniel Gruneberg, Julia Sander, Philipp Schulz, Martin Loos, Moritz S Bischoff, Lars Pursche, Markus A Weigand, Felix C. F. Schmitt

**Affiliations:** 1 Department of Anesthesiology, Medical Faculty Heidelberg, Heidelberg University, Heidelberg, DEU; 2 Institute of Medical Biometry and Informatics, Medical Faculty Heidelberg, Heidelberg University, Heidelberg, DEU; 3 Department of General, Visceral and Transplantation Surgery, Medical Faculty Heidelberg, Heidelberg University, Heidelberg, DEU; 4 Department of Vascular and Endovascular Surgery, Medical Faculty Heidelberg, Heidelberg University, Heidelberg, DEU; 5 Department of Urology, Medical Faculty Heidelberg, Heidelberg University, Heidelberg, DEU

**Keywords:** post-anesthesia care unit, anesthesia, patient safety, perioperative, monitoring system

## Abstract

Background: Medical research aims to improve patient safety and efficiency in the perioperative setting. One critical aspect of patient safety is the intrahospital transfer of patients. Also, reliable monitoring of vital signs is crucial to support the medical staff. This study was conducted to assess two monitoring systems in terms of the handover time and staff satisfaction.

Methods: To assess several aspects, two monitoring systems were compared: an organizational unit-related monitoring system that needs to be changed and brought back to the initial organizational unit after the patient transfer and a patient-specific monitoring system that accompanies the patient during the whole perioperative process.

Results: In total, 243 patients were included, and 375 transfers were examined to analyze economic factors, including differences in handover times and user-friendliness. To this end, 30 employees of the Heidelberg University Hospital were asked about their satisfaction with the two monitoring systems based on a systematic questionnaire. It could be shown that, especially during transfers from the operating theater to the intensive care unit or the recovery room, the time from arrival to fully centralized monitoring and the total handover time were significantly shorter with the patient-specific monitoring system (p < 0.001). Furthermore, the staff was more satisfied with the patient-specific monitor system in terms of flexibility, cleanability and usability.

Conclusion: The increased employee satisfaction and significant time benefits during intrahospital transports may increase patient safety and efficiency of patient care, reduce employee workload, and reduce costs in the overall context of patient care.

## Introduction

Patient safety is increasingly becoming the focus of daily clinical practice, especially in times of limited human resources. It is highly influenced by the qualifications and number of staff present and the quality of the equipment used [1]. Modern medicine is taking advantage of the technical progress and uses monitoring systems to provide increasingly reliable and practical methods of guiding patients safely through several treatment stages, such as induction of anesthesia, surgery, and the following intensive care treatment. Transport between these organizational units is associated with several risks for the patient (e.g., desaturation, hypotension). It has been shown that almost half of the patients who were transferred to an intensive care unit station experienced a deterioration in either respiratory or hemodynamic status during the transfer [2]. The number of events increases up to 44% in transfers of critically ill patients [3]. Furthermore, intrahospital transfers from the emergency department have been reported to have a 33% rate of unexpected events, such as hypoxemia, arrhythmias, or cardiopulmonary arrest during the transfers [4]. Patient safety is composed of several dimensions, whereby several human factors, such as time pressure, workload, emergency situations, and organizational issues, affect intraoperative patient safety [5]. Moreover, the US Food and Drug Administration (FDA) has registered another worrying trend of over 500 alarm-related patient deaths within five years in the USA. A study in an intensive care unit showed that alarm fatigue occurred in two-thirds of the alarms [6]. Therefore, it is essential to set the alarm limits sensibly to avoid desensitization of the medical team [7]. For this purpose, a monitoring system must be simple for the staff without diverting the focus too much on complex device settings. Furthermore, correctly cleaning the patient-related equipment, including the monitor system, is essential. Cleaning time can be a further time factor and can retain employees.

The assessment of a monitoring system for perioperative and critically ill patients in relation to patient safety, efficiency, and workflow remains an understudied area in current research. In particular, critically ill patients who require time-critical care could benefit from shorter transmission times by optimizing processes and shortening technically induced delays.

To investigate these aspects, two monitor systems were examined in more detail concerning economic issues such as handover time or the time necessary for cleaning and disinfection. Furthermore, staff satisfaction was investigated for both monitoring systems as a secondary endpoint.

## Materials and methods

Study design

This prospective, observational clinical study was approved by the Ethics Committee of the Medical Faculty of Heidelberg (trial code no. S-160/2020; German clinical trials register no. DRKS00021656). The study participants signed written informed consent and were enrolled from September 2020 to March 2021. The study period was composed of three phases. In the first phase, intrahospital transfers were recruited with the Dräger Infinity® Delta monitor system (Drägerwerk AG & Co. KGaA, Lübeck, Germany) in the old surgical hospital from September 2020 to October 2020. A phase with five months of interruption followed this. To allow the staff some time to familiarize themselves with the new infrastructure, this five-month wash-out period was established. In the third and last phase, from February 2021 to March 2021, the intrahospital transfers with the Philips IntelliVue X3 monitoring system (Philips Medizin Systeme Böblingen GmbH, Böblingen, Germany) were surveyed in the new building of the surgical clinic.

Inclusion and exclusion criteria

The inclusion criteria required patients to be at least 18 years old and to have undergone major abdominal, urological, or vascular surgery. Exclusion criteria were emergency patients admitted through the emergency department, infectious patients with cholera, diphtheria, hemorrhagic fever, meningoencephalomyelitis, query fever, rabies, tuberculosis, Koch’s disease, typhoid fever, chickenpox, and generalized zoster disease since this would have required additional protective measures.

Monitoring systems

Intrahospital transfers of the study patients were compared at the surgical clinic of Heidelberg University Hospital, with a focus on the two different monitoring systems. A maximum of up to three transfers per included patient were allowed to be observed. The surgical clinic of Heidelberg University Hospital was moved from its old location to the new one in October 2020. The old surgical clinic used the Dräger Infinity® Delta monitor system. This monitor system was organizational unit-related, which can also be used for transfers but needs to be changed and brought back to the initial organizational unit after the patient transfer. In contrast, the Philips IntelliVue X3 modular system introduced a patient-related monitoring system, i.e., a monitor that remained with the patient during the whole perioperative process. The system was introduced for the new surgical clinic, with the additional monitor stations Philips IntelliVue MX850 for all operating rooms and the intensive care unit and the Philips IntelliVue MX550 in all anesthesiology inductions and the post-anesthesia care unit. The Philips IntelliVue X3 patient-related monitor has a relatively smaller display; the vital signs are displayed in an expanded manner on the MX850 and MX550 monitors when IntelliVue X3 is connected to these devices.

Study endpoints

The primary endpoint of this study was to investigate whether a patient monitoring system that remains with the patient during the perioperative course provides an advantage over a monitoring system that belongs to an organizational unit. The times were compared from when the patient arrived at the place of destination until all vital signs were displayed on the central monitor of the new ward, as well as the total handover time. The study data were documented by an additional study team member, who attended the transfer to ensure that the transfer process was not influenced and the data were unbiased. The total transport time was deliberately not used because the transfer distances between the two buildings differed considerably. All other procedures were comparable between the two study sides (e.g., handover processes). Additionally, adverse events were studied, and staff satisfaction regarding the two monitor systems was measured as the secondary endpoint.

Satisfaction assessment

A systematic questionnaire was used for the stratification of staff satisfaction with the several monitoring systems. For employees, the inclusion criteria were age ≥18 years and familiarity with the monitoring systems. Refusal to participate in the study was evaluated as an exclusion criterion. The staff members were solely employees of the Heidelberg University Hospital. An odd Likert scale ranging from “strongly disagree” with a value of 1 to “strongly agree” with a value of 7 was used, and an even Likert scale ranging from “unlikely” with a value of 1 to “very likely” with a value of 10 was used for one question.

Statistical analysis

The Institute of Medical Biometry (Institut für Medizinische Biometrie, or IMBI) at the Heidelberg University Hospital performed a statistical analysis of the data with the programming language R (version 4.2.0). Descriptive data were reported with the mean, standard deviation, and interquartile range for continuous parameters and scores of absolute and relative frequencies for categorical variables. Descriptors were stratified by group. Differences in different endpoints between monitor systems were calculated using 95% confidence intervals. Potential differences related to endpoints between monitor systems were tested for ordinal data, such as scores using the Wilcoxon U test, categorical data using the chi-square test, and continuous data using the t-test. Possible differences in employee questions about the usefulness of the monitoring system and overall impression were tested between the two monitoring systems with the Wilcoxon U test and reported with the Hodges-Lehmann estimator and associated 95% confidence intervals. Due to the study’s design as an exploratory data analysis, all results of the statistical tests are considered descriptive statistics for hypothesis generation.

## Results

A total of 243 patients matched the study criteria and were included. Divided by department, 18 patients (7%) underwent vascular surgery, 75 (31%) patients underwent urological surgery, and 150 (62%) patients underwent visceral surgery. Additional demographic data of the included patients are shown in Table 1.

**Table 1:** Demographic data of all patients ASA, American Society of Anesthesiologists

Patient characteristics	Total (n = 243)
Age	
Mean	62
Standard deviation	14
Interquartile range	53–72
Sex	
Male	153 (63%)
Female	90 (37%)
ASA classification (n = 239)	
I	13 (5%)
II	114 (48%)
III	110 (46%)
IV	2 (1%)

A total of 375 transfers were surveyed in 243 patients. Of these, 228 transfers from the anesthesia induction room to the operating room were surveyed, and 147 transfers from the operating room to the post-anesthesia care unit or an intensive care unit were surveyed. More specifically, 185 transfers were included in the old surgical hospital using the organizational unit-related monitoring system, and 190 transfers were included in the new surgical hospital using the patient-related monitoring system. The demographic data of the two cohorts are included in Table 2.

**Table 2:** Demographics of the cohort transferred from the anesthesia induction room to the operating room ^t^Welch’s two-sample t-test ^a^Pearson’s chi-squared test *is used to represent statistical significance (p < 0.05). ASA, American Society of Anesthesiologists

Patient characteristics	Organizational unit-related monitoring system (n = 113)	Patient-related monitoring system (n = 115)	Total (n = 228)	p
Age				0.052^t^
Mean	63	60	61	
Standard deviation	14	14	14	
Interquartile range	55–73	51–69	53–72	
Sex				
Male	66 (58%)	77 (67%)	143 (63%)	0.182^a^
Female	47 (42%)	38 (33%)	85 (37%)	
ASA classification				
I	5 (5%)	7 (6%)	12 (5%)	0.501^a^
II	54 (49%)	55 (48%)	109 (49%)	
III	49 (45%)	52 (46%)	101 (45%)	
IV	2 (2%)	0 (0%)	2 (1%)	
Specialty department				
Vascular Surgery	12 (11%)	0 (0%)	12 (5%)	0.002^a,^*
Urology	35 (31%)	40 (35%)	75 (33%)	
Visceral Surgery	66 (58%)	75 (65%)	141 (62%)	
Organs involved during surgery				
Pancreas	27 (25%)	29 (25%)	56 (25%)	0.965^a^
Esophagus	4 (4%)	9 (8%)	13 (6%)	0.201^a^
Liver	7 (7%)	10 (9%)	17 (8%)	0.560^a^
Stomach	4 (4%)	6 (5%)	10 (5%)	0.606^a^
Intestine	18 (17%)	19 (16%)	36 (16%)	0.789^a^
Kidney	25 (24%)	26 (23%)	51 (23%)	0.863^a^
Vessels	13 (12%)	2 (2%)	15 (7%)	0.002^a,^*
Pre-existing conditions				
Cardiovascular	38 (34%)	29 (25%)	67 (30%)	0.150^a^
Liver	20 (18%)	25 (22%)	45 (20%)	0.443^a^
Kidney	30 (27%)	40 (35%)	70 (31%)	0.178^a^
Hypertension	57 (50%)	55 (48%)	112 (49%)	0.693^a^
Diabetes	26 (23%)	22 (19%)	48 (21%)	0.473^a^
Neurology	18 (16%)	20 (17%)	38 (17%)	0.767^a^

During transfers between the anesthesia induction room and the operating room with the organizational unit-related monitoring system, rapid intervention by the transport team due to a change in vital signs was necessary for 4% of transfers. At the same time, this was necessary for 1% of transfers with the patient-related monitoring system (p = 0.162). Bradycardia occurred during a transfer from the anesthesia induction room to the operating room with the patient-related monitoring system. With the organizational unit-related monitoring system, two cases of intervention performed on transport occurred due to relevant saturation drops, one case due to respiratory rate, one case due to hypotension, and one case due to altered capnography.

During the transfer from the anesthesia induction room to the operating room, 82% of patients monitored with the organizational unit-related monitoring system were intubated, whereas 99% of patients monitored with the patient-related monitoring system were intubated (p < 0.001). Once in the operating room, it took an average of 21 seconds with the organizational unit-related monitoring system and 77 seconds with the patient-related monitoring system for all vital signs to be displayed on the central operating room monitor (p < 0.001). Table 3 shows the individual time intervals of the two monitoring systems from arrival in the operating room to when all vital signs were displayed on the central operating room monitoring system.

**Table 3:** Time interval between arrival in the operating room and the display of all vital signs on the operating room monitor system ^*^Mann–Whitney’s U test

Display of all vital signs within (seconds)	Organizational unit-related monitoring system (n = 113)	Patient-related monitoring system (n = 115)	Total (n = 228)	p
1–60	82 (73%)	21 (18%)	103 (45%)	<0.001*
61–120	26 (23%)	59 (51%)	85 (37%)	
121–180	2 (2%)	25 (22%)	27 (12%)	
181–240	1 (1%)	5 (4%)	6 (3%)	
241–360	0 (0%)	4 (3%)	4 (2%)	
361–420	1 (1%)	0 (0%)	1 (0%)	
>421	0 (0%)	1 (1%)	1 (0%)	

The second cohort consisted of transfers from the operating room to the post-anesthesia care unit or an intensive care unit. Seventy-two transfers were performed with the organizational unit-related monitoring system and 75 with the patient-related monitoring system. Further characteristics can be found in Table 4.

**Table 4:** Demographic data of the cohort transferred from the operating room to the post-anesthesia care unit or to an intensive care unit ^t^Welch’s two-sample t-test ^a^Pearson’s chi-squared test * is used to represent statistical significance (p < 0.05). ASA, American Society of Anesthesiologists

Patient characteristics	Organizational unit-related monitoring system (n = 72)	Patient-related monitoring system (n = 75)	Total (n = 147)	p
Age				0.012^t,^*
Mean	65	60	62	
Standard deviation	15	13	14	
Interquartile range	58–77	51–69	54–73	
Sex				
Male	41 (57%)	52 (69%)	93 (63%)	0.119^a^
Female	31 (43%)	23 (31%)	54 (37%)	
ASA classification				
I	4 (6%)	5 (7%)	9 (6%)	0.532^a^
II	34 (48%)	36 (48%)	70 (48%)	
III	31 (44%)	34 (45%)	65 (45%)	
IV	2 (3%)	0 (0%)	2 (1%)	
Specialty department				
Vascular Surgery	12 (17%)	0 (0%)	12 (8%)	<0.001^a,^*
Urology	25 (35%)	27 (36%)	52 (35%)	
Visceral Surgery	35 (49%)	48 (64%)	83 (56%)	
Organs involved during surgery				
Pancreas	11 (15%)	13 (17%)	24 (16%)	0.764^a^
Esophagus	1 (1%)	2 (3%)	3 (2%)	0.592^a^
Liver	4 (6%)	9 (12%)	13 (9%)	0.177^a^
Stomach	2 (3%)	6 (8%)	8 (5%)	0.169^a^
Intestine	13 (18%)	16 (21%)	29 (20%)	0.647^a^
Kidney	19 (27%)	16 (21%)	35 (24%)	0.443^a^
Vessels	12 (17%)	2 (3%)	14 (10%)	0.004^a,^*
Pre-existing conditions				
Cardiovascular	27 (38%)	22 (29%)	49 (34%)	0.266^a^
Liver	11 (15%)	18 (24%)	29 (20%)	0.184^a^
Kidney	18 (25%)	25 (33%)	43 (29%)	0.267^a^
Hypertension	37 (51%)	29 (39%)	66 (45%)	0.121^a^
Diabetes	14 (19%)	11 (15%)	25 (17%)	0.441^a^
Neurology	12 (17%)	9 (12%)	21 (14%)	0.419^a^

From the operating room to the post-anesthesia care unit or intensive care unit, rapid intervention by the transport team due to a change in vital signs was required in 12% of transfers with the organizational unit-related monitoring system and in 7% of transfers with the patient-related monitoring system (p = 0.313). Saturation drops occurred in six transfers with the organizational unit-related monitoring system and three cases with the patient-related monitoring system (p = 0.569). The patient’s respiratory rate decreased on one transfer with the organizational unit-related monitoring system (p = 0.411). Both monitoring systems required blood pressure intervention. Thus, hypotension occurred in three transfers with the organizational unit-related monitoring system and one case with the patient-related monitoring system (p = 0.506). On five transfers from the operating room to a monitoring station with the organizational unit-related monitoring system, the patient was ventilated. On one transfer, there was an inadvertent disconnection from the ventilator.

In contrast, with the patient-related monitoring system, eight patients were transported ventilated to the monitoring station (p = 0.427). On a transfer from the operating room to a monitoring station, there was a problem with the monitor due to a malfunction in the organizational unit-related monitoring system (p = 0.302). There was a significant difference between the two monitoring systems in the time it took for all vital signs to be displayed on the central monitor once the patient arrived at the destination station. It took 241 seconds for the organizational unit-related monitoring system to display all vital signs and 98 seconds for the Philips IntelliVue X3 (p < 0.001). Table 5 shows the times broken down into one-minute intervals.

**Table 5:** Time intervals between the arrival in a monitoring area (e.g., recovery room, ICU, etc.) and the display of all vital signs on the fixed installed monitoring system ^t^Welch’s two-sample t-test

Display of all vital signs within (seconds)	Organizational unit-related monitoring system (n = 72)	Patient-related monitoring system (n = 75)	Total (n = 147)	p
1–60	2 (3%)	9 (12%)	11 (8%)	<0.001^t^
61–120	5 (7%)	33 (44%)	38 (26%)	
121–180	8 (12%)	19 (25%)	27 (19%)	
181–240	18 (26%)	7 (9%)	25 (17%)	
241–300	15 (22%)	5 (7%)	20 (14%)	
301–360	9 (13%)	1 (1%)	10 (7%)	
361–420	3 (4%)	1 (1%)	4 (3%)	
421–480	3 (4%)	0 (0%)	3 (2%)	
481–540	1 (1%)	0 (0%)	1 (1%)	
541–600	2 (3%)	0 (0%)	2 (1%)	
601–660	1 (1%)	0 (0%)	1 (1%)	
>661	2 (3%)	0 (0%)	2 (1%)	

To clean each organizational unit-related monitoring system after the transfer, the employees needed a mean time of 65 seconds, while the patient-related monitoring system stayed with the patient and was cleaned after discharge from the high-care unit. The purification of all dirty monitoring systems was performed once a day.

Thirty healthcare providers were enrolled to investigate staff satisfaction using a scaled questionnaire. Of the staff surveyed, 10% were nurses, and 90% were doctors. Further demographic data of the staff are shown in Table 6.

**Table 6:** Demographic data of the medical staff

Healthcare provider characteristics	Total (n = 30)
Age	
Mean	33
Standard deviation	5.3
Interquartile range	30–34
Sex	
Male	23 (77%)
Female	7 (23%)
How many years have you been in a clinical profession? (years)	
<1	1 (3%)
1 – <3	11 (37%)
3 – <5	6 (20%)
5 – <10	8 (27%)
>10	4 (13%)
How often do you use the transport system?	
Once per week	1 (3%)
Regularly, every few shifts	7 (23%)
Once per shift	1 (3%)
Multiple times per shift	21 (70%)

The employee survey showed a significant difference regarding the comparison of both monitor systems in the statements. The staff was more satisfied with the patient-specific monitor system, especially in terms of flexibility, cleanability, and usability. An overview of all questions is shown in Table 7.

**Table 7:** Results of the employee questionnaire regarding satisfaction with using the organizational unit-related monitoring system and the patient-related monitoring system The questionnaire consists of a 7-point Likert scale with 1 = “strongly disagree” to 7 = “strongly agree,” and a 10-point Likert scale with 1 = “unlikely” to 10 = “very likely”. Results are presented as averages, with the interquartile ranges in parentheses. ^a^On a 10-point Likert scale with 1 = "unlikely" to 10 = "very likely" ^b^Student’s paired t-test * is used to represent statistical significance (p < 0.05).

Question	Organizational unit-related monitoring system (n = 30)	Patient-related monitoring system (n = 30)	p
The transport monitor system...			
helps me be very effective	4.6 (4–6)	5.1 (4–6)	0.150^b^
helps me to be very productive	4.6 (4–6)	5 (4–6)	0.261^b^
is very useful	5 (4–6)	5.3 (5–6)	0.313^b^
gives me very reasonable control over the activities during my work	5.4 (5–6)	5.4 (5–6)	0.873^b^
makes it very easy to put activities I want to do into action	4.8 (4–6)	4.6 (4–5)	0.538^b^
I can operate without spending a lot of time	5 (4–6)	4.6 (4–6)	0.225^b^
fulfills my requirements	4.8 (4–6)	4.9 (4–6)	0.666^b^
does everything I would expect from it	5 (4–6)	5 (4–6)	>0.999^b^
It is very simple to use.	5 (4–6)	5 (4–6)	>0.999^b^
It is very easy to use.	4.8 (4–6)	5 (4–6)	0.617^b^
It is very user-friendly.	4.4 (3–5)	4.8 (4–6)	0.274^b^
It requires the least number of steps possible to accomplish what it wants to accomplish.	4.3 (3–5)	4.3 (3–6)	>0.999^b^
It is very flexible.	3.6 (3–5)	5.1 (4–6)	0.001^b,^*
Using it is effortless.	4.3 (3–5)	4.5 (4–6)	0.550^b^
I can use it without written instructions.	5.5 (5–6)	5.1 (4–6)	0.194^b^
I don’t notice any inconsistencies when I use it.	4.9 (4–6)	4.8 (4–6)	0.556^b^
Both regular and occasional users liked it.	4.5 (4–5)	4.7 (4–6)	0.541^b^
I can correct operating errors very quickly and easily.	4.7 (4–6)	4.5 (4–5)	0.556^b^
I can use it successfully at any time.	5.1 (5–6)	4.8 (4–6)	0.438^b^
I learned very quickly how to use it.	5.4 (5–6)	5.1 (4–6)	0.293^b^
I can very easily memorize how to use it.	5.4 (5–6)	5.1 (4–6)	0.500^b^
It is very simple to learn in its use.	5.3 (4–6)	5.1 (4–6)	0.536^b^
I quickly became proficient in the use of the transport monitor system.	5.3 (5–6)	5.3 (4–6)	>0.999^b^
I am very satisfied with the system.	4.6 (4–5)	5.1 (4–6)	0.270^b^
I would recommend it to a colleague.	4.4 (3–5)	5.2 (4–6)	0.043^b,^*
It’s fun to use.	3.6 (3–5)	4.2 (3–5)	0.202^b^
It works the way I would expect it to.	5 (4–6)	5 (4–6)	>0.999^b^
It’s great/super.	3.9 (3–5)	4.5 (4–6)	0.122^b^
I think every clinic should use it.	3.7 (3–5)	4.6 (3–6)	0.040^b,^*
It is very pleasant to use.	4 (3–5)	4.9 (4–6)	0.027^b,^*
There are always transport monitors available if needed.	5.5 (4–7)	5.6 (5–7)	0.908^b^
The transport monitor is very easy to clean.	4.9 (4–6)	5.6 (5–6)	0.030^b,^*
The transport monitor is very fast to clean.	4.8 (4–6)	5.5 (5–6)	0.017^b,^*
The transfer monitor can be attached so that it is secure/fixed to the bed during transfer.	4.9 (3–6)	4.2 (3–6)	0.171^b^
I have all the information I am interested in during the transfer.	5.7 (5–7)	5 (4–6)	0.083^b^
The transport monitor screen is adequate to be seen during intrahospital transport.	5.6 (5–7)	4.2 (3–5)	<0.001^b,^*
How likely would you recommend the transport monitor system to a colleague?^a^​​​​​	5.9 (4–7)	6.8 (6–8)	0.126^b^

## Discussion

This study compared two monitoring systems: a patient-related monitoring system and an organizational unit-related monitoring system. A significantly shorter handover time of perioperative patients occurred when using a patient-related monitoring system (p < 0.001). Additionally, the staff preferred the patient-related monitoring system in terms of flexibility (p < 0.001) and cleanability (p = 0.03 and p = 0.017), and they would recommend it to other colleagues (p = 0.043). However, the screen of the organizational unit-related monitoring system seemed to be better overviewed during patient transportation in the perioperative setting (p < 0.001).

Patient safety is one of the key factors in the perioperative setting and can be improved with modern monitoring systems. With the help of technological progress, such monitoring systems should enable medical personnel to identify critical situations early. In this study, the rate of adverse events in adult surgery patients in the perioperative setting ranged from 1% to 12%, depending on the transfer and the monitoring system used. Recent studies showed that a 17% to 50% rate of adverse events could be described for the intrahospital transfer of critically ill patients [2-4,8]. However, it is essential to note that the cohort of critically ill patients cannot be readily extrapolated to perioperative patients. For example, a study of pediatric surgery patients showed an incidence rate of only 3.4% for adverse events [9]. Since 53% of the patients in this study are ASA I and II patients and perioperative cohorts partly consist of critically ill patients, comparing perioperative and critically ill cohorts is delicate.

The central role of patient safety through monitoring systems can be illustrated, for example, by measuring pulsatile saturation. It can be used to identify a critical situation at an early stage and to treat it immediately. For example, with the introduction of oxygen saturation monitoring by pulsatile saturation measurement, a more than 10-fold reduction in anesthesia mortality was demonstrated in the USA between 1985 and 2000 [10,11]. Also, specific to the post-anesthesia care unit, studies have shown that the incidence of hypoxemia is reduced in patients in whom saturation is monitored compared to patients in whom medical staff could not see saturation levels [12].

The burden of intrahospital patient transfers on the medical staff is also demonstrated by a study that examined the time burden of intrahospital patient transfers on nurses. In a standard ward alone, 11.3 full-time equivalent nurses are needed per month just for intrahospital transfers [13]. Furthermore, a survey of intensive care nurses showed that 75% had indicated that intrahospital transfers resulted in an increased nurse workload [14].

The results were statistically significant between the two monitoring systems regarding the duration between the arrival at the target station and the moment until all vital signs were displayed on the monitor centrally held at the target station (p < 0.001). This time window was mostly comparable; any confounding factors, such as the distance from the operating room to the target station or the different complexities of the handover, can be neglected here. Significant differences were shown between the two monitoring systems during the transfer from the anesthesia induction room to the operating room until the vital signs were displayed entirely on the central monitor in the operating room. This could be because the patient-related monitoring system is usually appropriately positioned at the head end of the patient stretcher. At this point, the anesthesiologist is primarily concerned with connecting the patient to the ventilator. The organizational unit-related monitoring system either had to be carried in one hand or transported on the operating table in a poorly visible position and was presumably connected to the central monitoring system immediately and only then was the patient connected to the ventilator. During the transfer from the operating room to the monitoring area, the patient-related monitoring system was significantly superior to the organizational unit-related monitoring system, saving over two minutes at the destination station. This time advantage is doubly important, as both the transferring and receiving teams generate a time advantage. On average, for every 30 patients admitted, 60 minutes can be saved for both teams. For example, approximately 1 hour and 20 minutes can be saved per day in a hospital with eight operating theaters and five surgical procedures per room. Over a year with an average of 240 working days, this would result in 320 hours saved per year for the anesthesiology team alone. The same proportion of time can be saved for the recovery room or the intensive care unit team. Hence, it can be seen that a monitoring system that remains with the patient and does not need to be replaced leads to significantly faster processes. Additionally, the mean cleaning time of the organizational unit-related monitoring system was 65 seconds before it was ready to use for the following patient. In contrast, the patient-related monitoring system stayed with every patient and was cleaned after discharge from the high-care unit. To use the previous example, in a hospital with eight operating theaters, five surgical procedures per room, and 240 working days, a patient-related monitoring system would save approximately 43 minutes per day and 173 hours per year. That implication of similar monitoring devices was already tested and resulted in a higher cost-effectiveness and an increase in patient safety [15,16].

The employee survey also showed that the patient-related monitoring system is superior to the organizational unit-related monitoring system in terms of flexibility and cleaning. Tscholl et al. showed that 88% of staff agreed that “switching between monitors from different manufacturers is difficult,” so a patient-related monitor system passed on to every further organizational unit with a time handover advantage should still yield a clear benefit [17]. Furthermore, the colleagues surveyed would recommend it to other colleagues and clinics. Only the visibility of the patient-related monitoring system on intrahospital transfers was rated disadvantageously compared to the organizational unit-related monitoring system. It should be mentioned here that at the time of the study and with the opening of the new building for the surgical clinic, there were still no mounts for the patient-related monitoring system. Therefore, the visibility of the monitor systems at the patient’s bedside and, thus, during transfers from the operating room to a monitoring ward cannot be conclusively clarified.

This investigation acknowledges several limitations that could influence the findings. Primarily, the divergent infrastructural characteristics of the two buildings (old building of the surgical clinic, phase I, vs. new building of the surgical clinic, phase III) under study inherently affect the operational capacities of their respective monitoring systems. Consequently, these structural disparities introduce variables that may not be easily quantifiable, complicating direct comparisons. Furthermore, some points were difficult to quantify (e.g., visibility). These points were covered by the questionnaires, but maybe not in all possible aspects of the daily clinical routine (e.g., impact of environmental settings like darkness or bright light). Additionally, the study contrasts a monitoring system that has been in use for a long time with a more modern system. The differences in handling and user interfaces between these systems could introduce biases affecting their performance efficacy and technological reliability, potentially impacting the comparative analyses.

The study was able to show that a patient-related monitoring system can have a significant benefit for prompt patient care, by saving critical resources such as medical staff by reducing workload and handover time. Furthermore, a patient-related monitoring system provides overall staff satisfaction through flexibility and cleanability, and the staff recommends its usage to colleagues.

## Conclusions

Patient safety during intrahospital transfers needs to be improved continuously. The study of two monitoring systems showed significant time differences and that a patient-related monitoring system could be advantageous. For example, more than two minutes per transfer could be saved at the place of destination for transfers from the operating room to a monitoring station. A patient-related monitoring system seems beneficial and should be used to optimize handover time for all perioperative patients. Overall, the staff survey demonstrated the excellent flexibility and easy and quick cleaning capability of the patient-related monitoring system, leading to a recommendation of its use to other colleagues.
